# Inhibition of the SOS response by olivetol improves the efficacy of DNA-damaging antibiotics in *Escherichia coli*

**DOI:** 10.1038/s41429-026-00935-x

**Published:** 2026-06-04

**Authors:** Beste Şener, Barış Gökalsın

**Affiliations:** 1 Biology Department, Marmara University, Institute of Pure and Applied Sciences, Istanbul, Turkey; 2 Department of Biology, Marmara University, Faculty of Science, Istanbul, Turkey

**Keywords:** Antimicrobial resistance, Bacteriology, DNA damage response, Drug discovery

## Abstract

It is known that the rapid increase in antibiotic-resistant bacteria is reducing the effectiveness of existing antibiotics. The spread of resistant *Escherichia coli* strains has become a major concern in public health. Developing compounds that increase antibiotic activity is a promising alternative strategy to overcome this problem. SOS response mechanism in bacteria activates DNA repair and increases antibiotic resistance. Natural sources, such as lichens, are valuable for discovering potential SOS inhibitors due to their unique secondary metabolites with medicinal potential. This study aims to enhance the efficacy of antibiotics in *E. coli* by inhibiting SOS with olivetol, a natural metabolite derived from lichens. The SOS inhibition potential of olivetol was quantitatively assessed using qRT-PCR and GFP-based monitor strains. Moreover, MIC values of DNA damage-inducing ciprofloxacin, trimethoprim, nitrofurantoin and mitomycin C antibiotics were reassessed following combined treatment with olivetol. Overall, the results show that olivetol effectively suppresses the SOS response at high concentrations and reduces the MIC values of the antibiotics trimethoprim, nitrofurantoin, and mitomycin C. These findings indicate that olivetol has significant potential as a natural SOS inhibitor that can enhance the performances of certain antibiotics.

## Introduction

The therapeutic use of antibiotics to treat bacterial infections is becoming increasingly limited due to the rise in antibiotic resistance among bacteria. The detection of resistance to all antibiotics in clinical use poses a serious threat to global public health [1]. Moreover, it is known that at least 700,000 people die every year due to infections resistant to antimicrobial drugs. It is projected that this number could reach 10 million by 2050 and could be the main cause of death worldwide [2].

The treatment of *Escherichia coli* infections, which affect hundreds of millions of people each year, is threatened by the spread of resistant *E. coli* strains [3]. Pathogenic *E. coli* strains such as O157:H7 can cause serious illnesses such as severe hemorrhagic colitis, diarrhea, and hemolytic uremic syndrome by producing Shiga toxin [4]. Moreover, in the United States, more than 265,000 foodborne illness cases associated with Shiga toxin-producing *E. coli* (STEC) occur each year, resulting in 3600 hospitalizations and 30 deaths [5]. According to a report released by the European Centre for Disease Prevention and Control (ECDC) in 2019, 44% of bacterial infection cases identified in Europe were attributed to *E. coli*, and more than half of these cases were reported to be resistant to at least one antibiotic [6].

Therefore, there is a need to develop new alternative strategies to combat bacterial infections. One such strategy involves compounds that reduce resistance mechanisms without killing bacteria. For example, quorum sensing (QS) is a bacterial communication system that regulates virulence, biofilm formation, and resistance without inhibiting bacterial growth. QS inhibitors such as allicin [7], aloin, and emodin [8] block the QS system through various mechanisms, including inhibition of autoinducer synthesis, signal receptor antagonism, enzymatic degradation (quorum quenching), or suppression of gene expression regulated by QS [9]. Meanwhile, efflux mechanisms are considered a critical factor in the development of resistance to many antibiotics because they increase intracellular antibiotic concentrations by inhibiting the activity of membrane transporters responsible for the efflux of antibiotics in bacteria. Compounds such as reserpine [10], berberine [11], and piperine [12] inhibit efflux mechanisms, thereby restoring microbial susceptibility to antibiotics or reducing the opportunity for resistant mutants to be selected [13]. Another promising approach for reducing antibiotic resistance is the SOS response. Targets for inhibiting the SOS pathway include preventing the activation of the regulon by inhibiting RecA filamentation and blocking LexA autoproteolysis [14]. Compounds that possess SOS-inhibiting properties include suramin [15], zinc acetate [16]. In this context, one target has emerged: to enhance the efficacy of antibiotics that cause DNA damage by inhibiting the SOS response, which induced by these antibiotics and contributes to bacterial survival, mutagenesis and adaptive resistance, thereby reducing the development of antibiotic resistance.

**Fig. 1:**
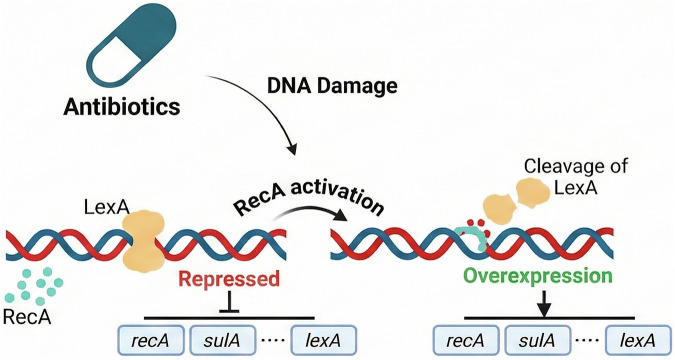
Activation of the SOS pathway

Originally known only to regulate DNA damage repair, the SOS response is a global stress response activated in both susceptible and resistant bacteria upon DNA damage and plays a role in physiological processes that enable bacteria to develop resistance to antibiotics [17, 18]. Antibiotics that cause DNA damage induce the SOS response, leading to detoxification of reactive oxygen species (ROS), horizontal gene transfer (HGT), biofilm formation, persister cell formation, hypermutation states, and the formation of small colony variants (SCVs) [19]. Therefore, inhibition of the SOS response has emerged as a rational adjuvant strategy. It has also reported that adaptive resistance mutations induced by antibiotic treatment and the acquisition of resistance genes are also due to the activation of the SOS system [20]. Bacteria exposed to various stresses, including antimicrobials that cause DNA damage, induce the expression of a set of genes known as the SOS response (Fig. 1). These genes are mainly regulated by two key proteins: LexA protein suppresses the response in the absence of DNA damage, whereas RecA protein activates the SOS system to express target genes when DNA damage occurs [21]. When DNA damage occurs, RecA binds to single-stranded DNA (ssDNA) forming an ssDNA-RecA complex (active RecA). It then triggers the autoproteolysis of LexA, inactivating it and inducing the expression of over 50 SOS-associated genes. These genes include *dinI*, a DNA damage-inducible protein that stabilizes RecA filaments, *sulA*, an inhibitor of cell division, *dinB* and *umuDC*, which encode low-quality and error-prone DNA polymerases with diverse functions such as DNA repair and mutagenesis [22].

Inhibition of the SOS response has become a target as a potential and promising treatment method to increase the sensitivity of existing antibacterial agents used today for bacterial infections. Bellio et al. (2017) demonstrated that certain secondary metabolites, such as lichesterinic, divaricatic, epiphorellic acids, tumidulin and sphaerophorin, inhibit the ATP-hydrolytic activity of RecA [23]. In addition, previous studies have shown that deletion of the *recA* gene restores sensitivity to ciprofloxacin in resistant *E. coli* strains [24] and that phthalocyanine tetrasulfonate (PcTs)-based chemical RecA inhibitors enhance the efficacy of antibiotic classes such as quinolones, β-lactams, and aminoglycosides by blocking the SOS response [25].

At this stage, the need to identify natural resources that can inhibit the SOS response emerges. With more than 20,000 species and covering 8% of the Earth’s surface, lichens have been used in the treatment of diseases for a long time [26]. Lichens have the unique ability to naturally synthesize many secondary metabolites in response to environmental stress. Among these metabolites, well-characterized compounds such as usnic acid, atranorin, fumaprotoketaric acid, and depsidones have been extensively studied and demonstrated for their various biological effects [26, 27]. The potential medical applications of secondary metabolites with varying functions and properties have motivated numerous researchers to investigate this subject. These metabolites, which have various biological activities such as antibacterial [28], antiviral [29], antifungal [30], antitumor [31] and anti-QS [32], have attracted considerable attention in antimicrobial research. Taken together, these metabolites may be promising candidates for the discovery of natural SOS inhibitors.

Olivetol, also known as 5-pentylresorcinol, is a naturally occurring metabolite found in lichens and is classified as a resorcinolic phenol. Additionally, olivetol is a precursor molecule for tetrahydrocannabinol (THC) and cannabidiol (CBD) in the biosynthesis of cannabinoids, particularly in the plant species *Cannabis sativa*. [33]. Olivetol is a naturally occurring metabolite found in several lichen species. It has been detected in *Cetrelia monachorum* and *Lepraria incana* [34], whose extracts show anti-inflammatory activity. It is also present in *Hypogymnia tubulosa* and *Hypogymnia physodes*, which exhibit antimicrobial and antifungal activities [26, 35]. In addition, Taslimi and Gulçin (2018) revealed in their study that olivetol has antioxidant and anticholinergic properties. Olivetol’s diverse biological activities, small molecular composition and affordability indicate that it could be a promising compound for targeting the SOS response [33].

This study aims to investigate the potential of olivetol, a lichen-derived secondary metabolite, as an inhibitor of the SOS response in *E. coli*. Although olivetol is known for various biological activities, its effect on the bacterial SOS response has not yet been explored. It is hypothesized that olivetol suppresses SOS activation and thereby enhances the efficacy of DNA-damaging antibiotics. This approach targets antibiotics that inhibit DNA replication and integrity, including ciprofloxacin (DNA gyrase and topoisomerase IV inhibitor), trimethoprim (indirectly suppresses DNA synthesis by affecting folate synthesis), nitrofurantoin (causing DNA damage by forming reactive intermediates) and mitomycin C (forming DNA cross-linking) [36].

To evaluate this potential, SOS green fluorescent protein (GFP) reporter assays were performed using *recA-gfp* and P*sulA-gfp* biomonitor strains. Subsequently, the minimum inhibitory concentration (MIC) values of the relevant antibiotics were determined. Effective olivetol concentrations identified in the reporter assays were then combined with antibiotics at sub-MIC levels to assess their effects on MIC values. Finally, qRT-PCR analysis was conducted to examine changes in the expression of the SOS-related genes *recA, sulA*, and *lexA*. Together, these approaches provide a comprehensive evaluation of whether olivetol can enhance antibiotic efficacy by targeting the SOS response.

## Materials and methods

### Bacterial strains

*E. coli* strains used in this study included *E. coli recA-gfp* (Pwt-*recA-gfp*; *recA-gfp* fusion with wild-type RecA promoter) and P*sulA-gfp* (Wild type promoter) biomonitor strains were used to detect SOS inhibition. These monitor strains were kindly provided by Andrew Robinson and Antoine van Oijen from University of Wollongong [37, 38]. *E. coli* O157:H7 strain was used for antimicrobial tests and qRT-PCR analyses. Bacteria were normally grown in Tryptic Soy Broth (TSB), and Mueller-Hinton Broth (MHB) media were used for antimicrobial tests. For GFP expression assays, M9 minimal media supplemented with MgSO4 (2 mM), CaCl2 (0.1 mM), thiamine (2.5 mg/l), glucose [0.5% (wt/vol)], and casamino acids [0.5% (wt/vol)] was used. Kanamycin (25 µg/ml) was added to MHB and M9 media for biomonitor strains.

### SOS GFP reporter assay

A modified method of Henrikus et al. (2020) was utilized for GFP expression assays [39]. GFP fluorescence measurements were performed in 96-well black microplates containing M9 growth medium. These media contained ciproloxacin with a final concentration of 0.002 µg/ml, which is necessary for SOS activation. *recA-gfp* and P*sulA-gfp* biomonitor strains were treated with olivetol ( ≥ 98% purity; Sigma-Aldrich, St. Louis, MO, USA), which was dissolved in dimethyl sulfoxide (DMSO), at final concentrations of 400 μM and 800 μM, respectively, to reach a total culture volume of 200 μl with an OD_600_ of 0.1. To allow for a clearer monitoring of the time dependent changes in GFP fluorescence, the plates were incubated at 36 °C to slightly slow bacterial growth and plates were monitored every 15 minutes for 16 h using a multimode microplate reader (Biotek, USA). To minimize potential absorbance interference that could arise from intrinsic color of olivetol during GFP expression measurements, bacterial growth and was measured at OD_450_ nm; fluorescence measurements were performed at *λ*_excitation_ = 485 nm, *λ*_emission_ = 535 nm wavelengths. Inhibition percentages for the applied olivetol concentrations were calculated at the end of 16 hours.

### Minimum inhibitory concentration (MIC) assays

MIC values of ciprofloxacin, trimethoprim, nitrofurantoin and mitomycin C antibiotics were determined using broth microdilution method in accordance with CLSI guidelines (CLSI M07-A11). MHB medium was added to each well in 96-well plates and serial dilutions of the antibiotics were performed. *E. coli* O157:H7 and ATCC 25922 suspension was then added to obtain a final OD_600_ of 0.01, and plates were incubated at 37 °C with shaking. Bacterial growth was determined by absorbance measurements at OD_450_ nm, to minimize any potential absorbance interference caused by the characteristic color of the antibiotics, after 20 h using Cytation 3 multimode microplate reader. MIC values were recorded as the lowest antibiotic concentration at which no visible growth was observed, and for OD values showed ≥90% inhibition compared to the untreated control. Additionally, to clarify MIC values and distinguish them from the minimum bactericidal concentration (MBC), 10 µl of suspension taken from clear wells was spread onto Mueller–Hinton Agar (MHA) medium, and the plates were incubated at 37 °C for 24 h. Concentrations at which colony formation was observed on the agar surface were recorded as MIC, while concentrations at which no colonies were observed were recorded as MBC.

### Determination of antibacterial properties of olivetol

The antibacterial activities of 800 and 400 µM olivetol concentrations of secondary metabolite on O157:H7 and ATCC 25922 *E. coli* strains were determined. The experiments were carried out according to the method described in MIC methods using 96-well transparent microplates. After incubation at 37 °C with shaking at 160 rpm for 20 h, OD_450_ nm was measured using a Cytation 3 multimode microplate reader to assess the effect of olivetol on bacterial growth.

### Combination treatment assays

Experiments were performed according to a modified method of that described by Revitt-Mills et al. [38]. Olivetol at 800 μM and 400 μM concentrations were treated with ½×, ¼× and ⅛× MIC (sub-MIC1, sub-MIC2, sub-MIC3) of ciprofloxacin, trimethoprim nitrofurantoin and mitomycin C antibiotics to determine their combined antimicrobial activities. Experiments were conducted at 37 °C in MHB medium with shaking using *E. coli* O157:H7 and ATCC 25922 strains in 96-well transparent microplates. The final bacterial concentration was adjusted to an OD_600_ of 0.01, and changes in antibiotic MIC values were determined by comparing OD_450_ measurements after 20 h. New MIC values were defined as the lowest antibiotic concentration that completely inhibited visible bacterial growth and reduced optical density by at least 90% compared to the untreated control, as described in MIC assays. The suspension from the wells where no reproduction was observed was inoculated into MHA medium and MBC values were determined.

### Gene expression analyses

Gene expression analyses were performed according to a modified method of that described by Gökalsın et al. [40]. Overnight cultures of *E. coli* O157:H7 strain were treated with the 800 and 400 μM concentrations of olivetol. Additionally, ciprofloxacin was used to activate the SOS system at a final concentration of 0.002 µg/ml. Cultures were incubated at 37 °C until late log phase. After incubation, pellets were retrieved and bacterial RNA extraction was performed using Roche High Pure RNA Isolation Kit (Roche) along with random and oligo dT primers (kit components). Purities and concentrations of RNA samples were measured using Cytation 3 with Take3 plate (Biotek). cDNA synthesis of RNA samples whose concentrations were equalized was performed with Transcriptor First Strand cDNA Synthesis Kit (Roche). FastStart Essential DNA Green Master Kit (Roche) was used to measure the level of SOS genes, including *recA, sulA* and *lexA*. The primer sequences are listed in Table 1. Finally, the qRT-PCR findings were analyzed by Pfaffl method [41] and 16S gene was used as control for normalization. Expression levels were compared to the control group containing 1% DMSO.

**Table 1:** Primers used in qRT-PCR for *E. coli*

Primer	Direction	Sequence (5′ → 3′)	Reference
*recA*	Forward	AGATCCTCTACGGCGAAGGT	[48]
	Reverse	CCTGCTTTCTCGATCAGCTT	[48]
*sulA*	Forward	GCCGGGCTTATTCAGTGAAGT	[49]
	Reverse	CCTGAACCCATTCCCGACTC	[49]
*lexA*	Forward	GACTTGCTGGCAGTGCATAA	[48]
	Reverse	CGTAAGGGCCATGATGACTT	[48]
16S	Forward	CAGCTCGTGTCGTGAGATGT	[50]
	Reverse	CGTAAGGGCCATGATGACTT	[50]

### Statistical analyses

All experiments were carried out in triplicate. The statistical analyses were performed using GraphPad V 9.0 Prism statistics software. Data are presented as means; error margins are presented as standard deviation (SD). Statistical significance was determined using the Kruskal- Wallis test followed by Dunn’s multiple comparison test. For qRT-PCR analyses, statistical significance was assessed using the Mann–Whitney U test. *p* values below 0.05 were considered statistically significant. In the graphs, ns is shown as nonsignificant, *p < 0.05, **p < 0.01, ***p < 0.001 and ****p < 0.0001.

## Results

### Dose-dependent inhibitory effect of olivetol against *E. coli* SOS response

**Fig. 2:**
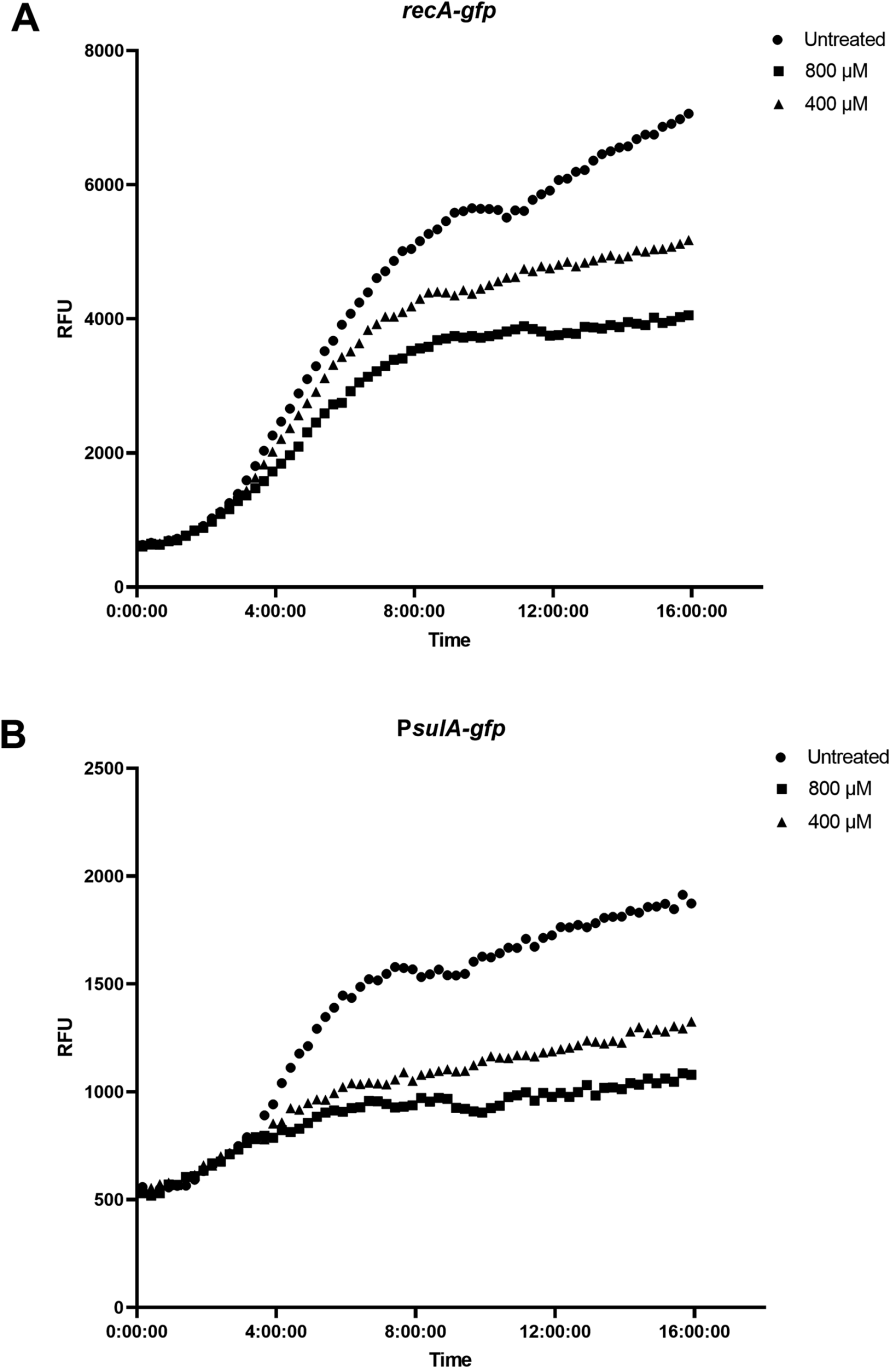
Dose–response curves showing the effects of olivetol treatment at 800 and 400 μM on *recA-gfp* (**A**) and *PsulA-gfp* (**B**) reporter strains. Data are presented as relative fluorescence units (RFU). No growth inhibition was observed at optical densities above 450 nm

The results, shown in Fig. 2, are the means of three replicates of GFP expressions. The results for the *recA-gfp* and P*sulA-gfp* strains are presented as relative fluorescence units (RFU) since olivetol treatment did not cause any growth inhibition on the reporter strains. SOS response inhibitions by olivetol were evaluated by comparison to untreated controls. Inhibition values were calculated based on RFU values measured after 16 h. The results indicate that olivetol suppresses GFP expression associated with *recA-gfp* and P*sulA-gfp*, achieving approximately 42.77 ± 2.03% and 43.85 ± 0.99% inhibition, respectively, at a concentration of 800 µM. 400 μM olivetol exhibited inhibition rates of 27.20 ± 2.04% for *recA-gfp* and 34.23 ± 2.15% for P*sulA-gfp*.

### MIC values of antibiotics

The ability of ciprofloxacin, nitrofurantoin, trimethoprim, and mitomycin C to cause DNA damage and thereby activate SOS system is widely known. Therefore, these antibiotics were selected to inhibit SOS response system with olivetol and thus reduce MIC values. In line with this objective, their MIC and sub-MIC values were determined using broth microdilution method for *E. coli* O157:H7 and ATCC 25922 strain. After incubation, untreated controls showed normal bacterial growth, while increasing antibiotic concentrations resulted in decreased turbidity and OD_450_ values. Ciprofloxacin exhibited the lowest MIC value (0.016 µg/ml) against both strains, whereas nitrofurantoin showed the highest MIC value at 14 µg/ml. Trimethoprim and mitomycin C MIC values of 0.25 µg/ml and 0.5 µg/ml, respectively. Subsequently, sub-MIC concentrations (½×, ¼×, and ⅛× MIC) were selected for olivetol treatment.

### Antibacterial properties of olivetol

**Fig. 3:**
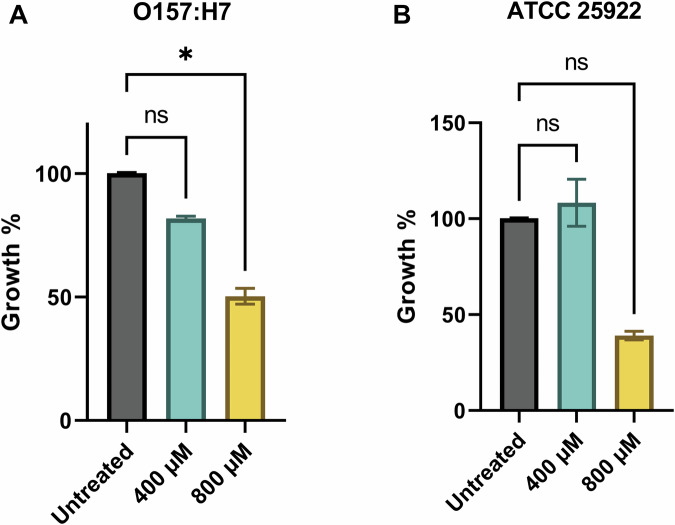
Percentage growth of *Escherichia coli* O157:H7 (**A**) and ATCC 25922 (**B**) strains following treatment with olivetol. Bacterial growth was monitored at OD₄₅₀ using a microplate reader. Growth was normalized to the untreated control and expressed as percentage growth inhibition. Olivetol at 800 μM showed a moderate inhibitory effect, whereas 400 μM had a minimal effect on cell growth. Data are presented as mean ± standard deviation (SD), statistical comparisons were performed relative to the untreated control

Olivetol secondary metabolite concentrations of 800 and 400 µM were applied to O157:H7 and ATCC 25922 strains and their antibacterial activities were tested. The results were analyzed by calculating the % inhibition values of bacterial growth at OD_450_ compared to the untreated control group. Growth % bars are presented in Fig. 3. Treatment with 800 µM olivetol against O157:H7 strain resulted in a 49.72 ± 4.13% inhibition in bacterial growth (*p* < 0.05). 400 µM olivetol showed no statistically significant difference, with an inhibition rate of 18.35 ± 2.60%. For the ATCC 25922 strain, 800 µM olivetol reduced bacterial growth by 63.68 ± 3.25%, while 400 µM olivetol increased bacterial growth by −7.37 ± 7.04%.

### Assessment of olivetol-induced potentiation of antibiotic activity

**Fig. 4:**
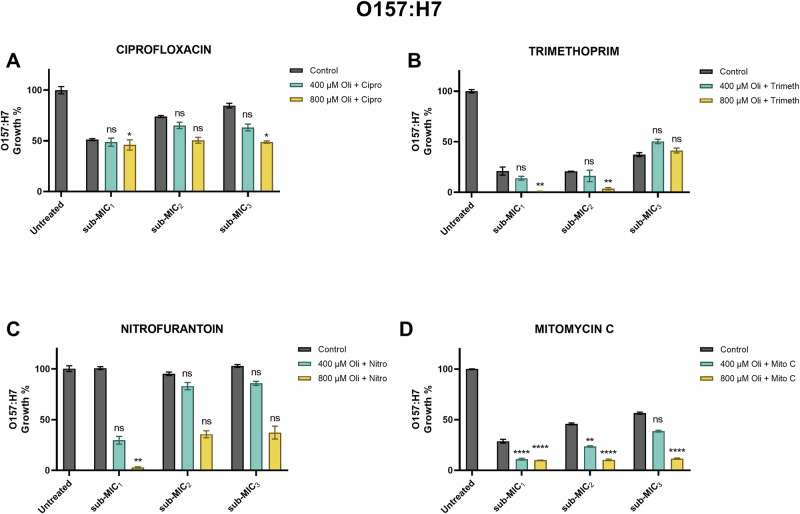
Inhibition percentages of olivetol–antibiotic combinations against *E. coli* O157:H7. The antibiotics used were **A**: ciprofloxacin (Cipro), **B**: trimethoprim (Trimeth), **C**: nitrofurantoin (Nitro), and **D**: mitomycin C (Mito C). Bacterial growth at OD₄₅₀ was analyzed and expressed as percentage inhibition. Bars represent growth under three sub-MIC antibiotic levels (sub-MIC1 = ½×MIC, sub-MIC2 = ¼×MIC, sub-MIC3 = ⅛×MIC), with or without olivetol. Untreated and antibiotic-only sub-MIC groups were included as controls. Growth in the presence of 400 or 800 μM olivetol is shown relative to antibiotic treatment alone. Data are presented as mean ± standard deviation (SD), statistical comparisons were performed relative to the untreated control

**Fig. 5:**
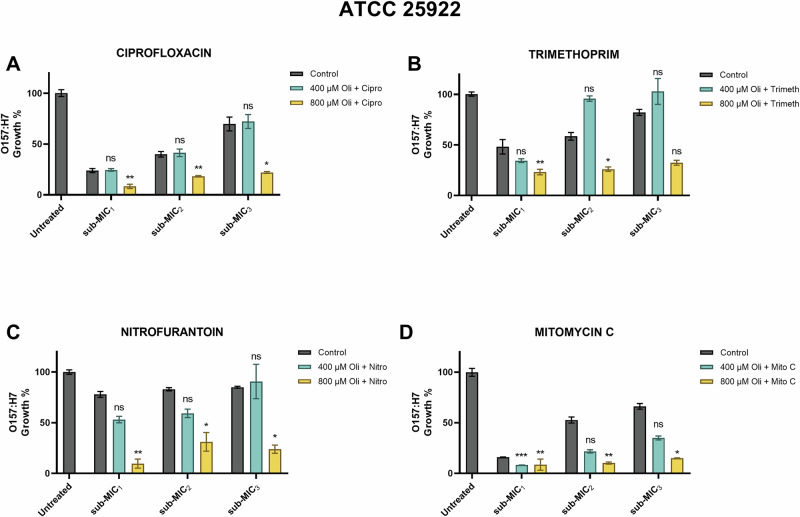
Inhibition percentages of olivetol–antibiotic combinations against *E. coli* ATCC 25922. The antibiotics used were **A**: ciprofloxacin (Cipro), **B**: trimethoprim (Trimeth), **C**: nitrofurantoin (Nitro), and **D**: mitomycin C (Mito C). Bacterial growth at OD₄₅₀ was analyzed and expressed as percentage inhibition. Bars represent growth under three sub-MIC antibiotic levels (sub-MIC1 = ½×MIC, sub-MIC2 = ¼×MIC, sub-MIC3 = ⅛×MIC), with or without olivetol. Untreated and antibiotic-only sub-MIC groups were included as controls. Growth in the presence of 400 or 800 μM olivetol is shown relative to antibiotic treatment alone. Data are presented as mean ± standard deviation (SD), statistical comparisons were performed relative to the untreated control

The DNA-damaging antibiotics ciprofloxacin, trimethoprim, nitrofurantoin and mitomycin-c were used in combination with olivetol. The results are shown in Figs. 4 and 5, and changes in MIC values are shown in Table 2. According to the results, olivetol at a concentration of 800 µM showed significant effects on trimethoprim, nitrofurantoin and mitomycin C in the O157:H7 strain. 800 µM olivetol concentration successfully halved the MIC of nitrofurantoin (*p* < 0.01) while reducing the MIC of mitomycin C by 8-fold (*p* < 0.0001). On the other hand, the combination of 800 µM olivetol + sub-MIC1 trimethoprim exhibited bactericidal activity. Furthermore, an olivetol concentration of 400 µM was sufficient to reduce the mitomycin C MIC value of the O157:H7 strain by a factor of 2 (*p* < 0.01). However, our tests showed that the ciprofloxacin MIC value remained unchanged in the presence of any olivetol concentration for O157:H7 strain. However, in the ATCC 25922 strain, 800 µM olivetol reduced the MIC of ciprofloxacin 2-fold (*p* < 0.01). Similarly, it halved the MIC of nitrofurantoin (*p* < 0.01) and showed the most pronounced effect against mitomycin C. While 400 µM olivetol reduced the mitomycin C MIC value 2-fold (*p* < 0.001), 800 µM resulted in a 4-fold reduction (*p* < 0.01). Additionally, although no change was observed in the MIC of trimethoprim, the combination of 800 µM olivetol and sub-MIC₁ trimethoprim reduced bacterial growth by 76.89 ± 2.71% (*p* < 0.01).

**Table 2:** Changes in MIC values (µg/ml) in *E. coli* O157:H7 and ATCC 25922 strains in the presence of olivetol

		MIC (µg/ml)			
		Ciprofloxacin	Trimethoprim	Nitrofurantoin	Mitomycin C
**O157:H7**	800 µM Olivetol	0.016	0.0625 (**)	7 (**)	0.0625 (**)
	400 µM Olivetol	0.016	0.25	14	0.125 (****)
	0 µM Olivetol	0.016	0.25	14	0.5
**ATCC 25922**	800 µM Olivetol	0.008 (**)	0.25	7 (**)	0.0125 (*)
	400 µM Olivetol	0.016	0.25	14	0.25 (***)
	0 µM Olivetol	0.016	0.25	14	0.5

Bold values indicate changes in MIC compared with the corresponding untreated control (0 µM olivetol)

Asterisks indicate the level of statistical significance: *p < 0.05, **p < 0.01, ***p < 0.001, ****p < 0.0001

### Olivetol downregulates the expression of SOS response-associated genes (*recA, sulA, lexA*)

**Fig. 6:**
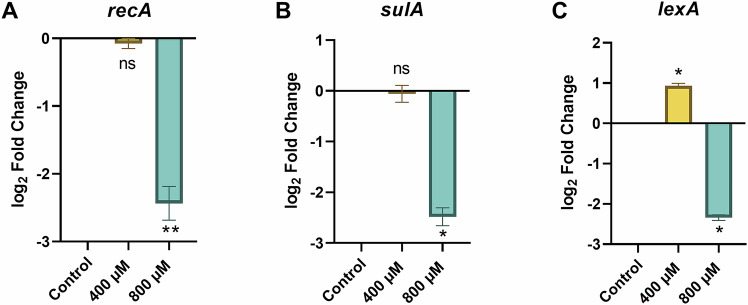
Relative expression levels of *recA* (**A**), *sulA* (**B**), and *lexA* (**C**) genes in *E. coli* O157:H7treated with 400 or 800 μM olivetol. Gene expression levels were normalized to 16S rRNA and compared with the 1% DMSO control group

The expression levels of the *recA, lexA* and *sulA* genes were relatively quantified. Cq values were analyzed using Pfaffl method, taking into consideration PCR efficiency and shown in Fig. 6. The results show that 800 μM olivetol substantially suppressed the expression levels of the *recA*, *sulA* and *lexA* genes by 83.24 ± 5.97%, 83.11 ± 5.59% and 57.28 ± 2.34, respectively. In contrast, treatment with 400 μM olivetol did not cause a statistically significant difference in *recA* and *sulA* expression compared to the control group, although it caused an upregulation in *lexA* expression. These findings indicate that olivetol can significantly suppress the SOS response in *E. coli* O157:H7, particularly at high concentrations.

## Discussion

In the near future, it is predicted that current antibiotics will become ineffective against increasing levels of bacterial resistance, and classic bacterial infections will re-emerge as major causes of mortality [1]. In particular, the spread of resistant strains of *E. coli*, which is the cause of common bacterial infections, has become a serious public problem. Accordingly, it is important to develop therapeutic interventions and alternative strategies to prevent and control these infections. Therefore, effective management of antibiotic resistance requires the development of compounds that either serve as alternatives to conventional antibiotics or enhance the efficacy of existing antibiotic therapies. In this context, the need for a better understanding of the relevant bacterial physiological pathways has become apparent. Particularly, SOS pathway functions in response to DNA damage and initiates various pro- survival and resistance mechanisms such as DNA repair, hypermutation and HGT. detoxification of ROS, biofilm formation, dissemination of virulence factors, and the production of SCVs. This may represent the SOS response a potential target for combating drug-resistant pathogens [42]. In this context, suppression of the SOS response is considered an important target for enhancing the efficacy of antibiotics and providing an effective strategy against antibiotic resistance [43]. However, despite the growing interest in targeting this pathway, the SOS inhibitory potential of many natural compounds, particularly lichen-derived secondary metabolites, remains largely unexplored. The present study demonstrates that the lichen metabolite olivetol inhibits the *E. coli* SOS response and enhances the efficacy of various antibiotics when used in combination.

According to reporter assay results, olivetol at 800 μM was significantly more effective than at 400 μM in inhibiting *recA* and *sulA* expressions. *recA-gfp* monitor strain was used because RecA functions as the activator of the SOS mechanism, whereas the P*sulA-gfp* monitor strain was utilized since *sulA* is directly regulated by the SOS response. The inhibition observed in *recA* and *sulA* in the GFP expression assays was consistent with the qRT-PCR results, which confirmed that 800 μM olivetol downregulated *recA* and *sulA* by approximately 6-fold.

These findings align with the known role of RecA in mediating DNA damage induced LexA autoproteolysis. By suppressing *recA* expression, olivetol is likely to stabilize LexA, thereby reducing the transcription of genes regulated by SOS [44]. The marked decrease in *sulA* expression, a gene directly regulated by LexA, supports the effective suppression SOS system [45]. The reduction in *lexA* mRNA is interpreted as olivetol increasing the stability of the LexA protein and downregulating gene expression through negative feedback. As long as the LexA protein is stable, the cell is thought to limit LexA production. Alternatively, it could be proposed that olivetol acts on another mechanism regulating *lexA* transcription [46].

Overall, the coordinated downregulation of *recA*, *sulA*, and *lexA* in the presence of 800 μM olivetol demonstrates that olivetol effectively suppresses activation of the SOS response. This finding agrees with previous studies showing that inhibition or silencing of *recA* suppresses SOS induction. Bellio et al. screened 27 lichen-derived secondary metabolites and identified nine compounds with over 80% RecA inhibition, highlighting lichens as a rich source of natural SOS inhibitors [23].

Since activation of the SOS system contributes directly to adaptive resistance, mutagenesis, and antibiotic treatment failure, targeting this pathway represents an effective strategy for improving antimicrobial activity [25, 43]. Results for the *E. coli* O157:H7 and ATCC 25922 strains demonstrated that olivetol molecule can reduce bacterial growth when used in combination with antibiotics that cause DNA damage. Consistent with gene expression and GFP reporter data, an olivetol concentration of 800 µM was more effective in reducing antibiotic MIC values for O157:H7 strain.

Among the tested antibiotics, mitomycin C exhibited the most pronounced efficacy against both *E. coli* O157:H7 and ATCC 25922 strains. MIC of mitomycin C for O157:H7 decreased 8-fold in the presence of 800 µM olivetol, whereas for ATCC 25922, it decreased 4-fold. Notably, even at a lower concentration (400 µM), olivetol reduced the MIC of mitomycin C by 2-fold for both strains. These findings are consistent with previous reports showing that disruption of the SOS response significantly increases mitomycin C activity. Mo et al. (2016) reported a 16-fold reduction in the MIC of mitomycin C in a Δ*recA* strain, suggesting that the complete elimination of *recA* leads to a stronger senitization effect than chemical inhibition alone [43].

In the case of trimethoprim for O157:H7 strain, a bactericidal effect was exhibited by 800 µM olivetol when combined with ¼×MIC of trimethoprim, further supporting the role of SOS suppression in enhancing antibiotic efficacy. In addition, while 800 µM olivetol successfully reduced the MIC of trimethoprim by 2-fold, no change in the MIC value was observed in the ATCC 25922 strain. Similarly, a previous study demonstrated that *recA* inactivation reduced the MIC values of trimethoprim and, alongside it, ceftazidime, fosfomycin, and colistin in *E. coli*, and limited the emergence of resistant mutants by blocking the SOS response [47].

For nitrofurantoin, treatment with 800 µM olivetol resulted in a 2-fold reduction in MIC values for both *E. coli* strains. In line with this observation, Mo et al. (2016) reported a ≥4-fold decrease in nitrofurantoin MIC Δ*recA* strains, further supporting the contribution of SOS inhibition to increased antibiotic susceptibility [43].

Conversely, the impact of olivetol on ciprofloxacin activity in the O157:H7 strain was not statistically significant, and no reduction in MIC values was observed. However, in the ATCC 25922 strain, the MIC value of ciprofloxacin was reduced by half in the presence of 800 µM olivetol. Previous studies have demonstrated that *recA* deletion can restore sensitivity to ciprofloxacin in resistant *E. coli* strains [24] and that chemical *recA* inhibitors enhance the activity of quinolones, β-lactams, and aminoglycosides by blocking the SOS response [25]. In contrast, the absence of any statistically significant effect observed in O157:H7 strain may reflect strain-specific differences, partial SOS inhibition, or fundamental differences between genetic deletion and chemical modulation of the *recA* gene. It is thought that the more pronounced reductions in MIC values reported in studies employing *recA* deletion are due to the complete elimination or silencing of the *recA* gene.

Collectively, these findings indicate that chemical suppression of the SOS response by olivetol partially mimics the effects of *recA* inactivation, thereby preventing RecA filamentation and leading to enhanced susceptibility to DNA damage-inducing antibiotics, although the magnitude of this effect varies depending on the antibiotic and bacterial strain.

Although SOS activation has been linked to biofilm formation, toxin production, and virulence regulation [42]; this study focused on the effects of olivetol on the SOS response and antibiotic susceptibility. Furthermore, the significant growth inhibition observed in combination therapies limits the evaluation of features associated with biofilm formation or virulence. To investigate these effects in detail, future studies conducted at lower sub-MIC levels would be valuable.

Nevertheless, several limitations should be considered. The potential of olivetol to reverse existing resistance could not be directly assessed. Although SOS inhibition is associated with reduced mutagenesis and resistance development, this study did not include long-term evolutionary analyses to evaluate olivetol’s effect on the emergence of resistance. To fully assess olivetol’s translational potential as an antimicrobial adjuvant, future studies incorporating in vivo evaluation, toxicological profiling, and long-term evolutionary analyses are required.

This study demonstrates that olivetol, at higher concentrations, can suppress the expression of SOS associated genes, leading to enhanced susceptibility of *E. coli* to DNA damage-inducing antibiotics, including ciprofloxacin, trimethoprim, nitrofurantoin, and mitomycin C. These findings highlight the potential of natural compound-based SOS inhibitors to improve antibiotic efficacy and limit antimicrobial resistance.
